# Qualitative Study of Pediatric Early Warning Systems’ Impact on Interdisciplinary Communication in Two Pediatric Oncology Hospitals With Varying Resources

**DOI:** 10.1200/GO.20.00163

**Published:** 2020-07-16

**Authors:** Dylan Graetz, Erica C. Kaye, Marcela Garza, Gia Ferrara, Mario Rodriguez, Dora Judith Soberanis Vásquez, Alejandra Méndez Aceituno, Federico Antillon-Klussmann, Jami S. Gattuso, Belinda N. Mandrell, Justin N. Baker, Carlos Rodriguez-Galindo, Jennifer W. Mack, Asya Agulnik

**Affiliations:** ^1^ St. Jude Children’s Research Hospital, Memphis, TN; ^2^ Unidad Nacional de Oncología Pediátrica, Guatemala City, Guatemala; ^3^ Francisco Marroquin University School of Medicine, Guatemala City, Guatemala; ^4^ Dana Farber Cancer Institute, Boston Children’s Hospital, Boston, MA

## Abstract

**PURPOSE:**

Hospitalized pediatric oncology patients are at high risk of deterioration and require frequent interdisciplinary communication to deliver high-quality care. Pediatric early warning systems (PEWS) are used by hospitals to reduce deterioration, but it is unknown how these systems affect communication about patient care in high- and limited-resource pediatric oncology settings.

**METHODS:**

This qualitative study included semistructured interviews describing PEWS and subsequent team communication at 2 pediatric cancer centers, 1 in the United States and 1 in Guatemala. Participants included nurses, and frontline and intensive care providers who experienced recent deterioration events. Transcripts were coded and analyzed inductively using MAXQDA software.

**RESULTS:**

The study included 41 providers in Guatemala and 42 providers in the United States (33 nurses, 30 ward providers, and 20 pediatric intensive care providers). Major themes identified include “hierarchy,” “empowerment,” “quality and method of communication,” and “trigger.” All providers described underlying medical hierarchies affecting the quality of communication regarding patient deterioration events and identified PEWS as empowering. Participants from the United States described the algorithmic approach to care and technology associated with PEWS contributing to impaired clinical judgement and a lack of communication. In both settings, PEWS sparked interdisciplinary communication and inspired action.

**CONCLUSION:**

PEWS enhance interdisciplinary communication in high- and limited-resource study settings by empowering bedside providers. Traditional hierarchies contributed to negative communication and, in well-resourced settings, technology and automation resulted in lack of communication. Understanding contextual elements is integral to optimizing PEWS and improving pediatric oncology outcomes in hospitals of all resource levels.

## INTRODUCTION

Caring for hospitalized pediatric oncology patients requires cohesive interactions among the patient, family, and interdisciplinary health care professionals.^1^ Effective communication decreases mortality rates, increases quality of care, and improves patient-centered outcomes.^2^ Conversely, loss of communication can result in serious patient harm. A 2015 review of sentinel safety events in the United States reported communication as the root cause in almost 80% of cases.^3^

**CONTEXT**

**Key Objective**
To evaluate how pediatric early warning systems (PEWS) affect interdisciplinary communication regarding hospitalized pediatric oncology patients in settings of varied resource levels.
**Knowledge Generated**
Qualitative analysis of 83 participant interviews demonstrated that PEWS enhance interdisciplinary communication by empowering pediatric providers in high- and limited-resource settings. However, elements including technology and medical hierarchies affect the quality, tone, and method of interdisciplinary communication.
**Relevance**
PEWS improve interdisciplinary communication in both high- and limited-resource pediatric settings and encourage providers to consider specific contextual elements to optimize the impact of PEWS on patient care.

Interdisciplinary team communication is most critical when resources are limited and morbidity and mortality rates are high. The pediatric oncology population is particularly vulnerable because of disease complications and treatment toxicity. These patients have a high rate of clinical deterioration, with up to 40% of pediatric oncology patients requiring intensive care during cancer therapy.^4^ Successful team communication about deteriorating patients allows providers to function within their scope of practice, minimizing duplicated work and wasted effort. Trust and positive social relationships have been associated with improved performance and higher-quality care in low- and middle-income countries (LMICs).^5^ Unfortunately, medical hierarchies, often entrenched in LMICs,^6^ interfere with team communication^7^ and may hinder safe patient care.

In addition to interdisciplinary cancer care, global safety research has focused on teamwork and communication related to resuscitation, end of life, and intensive care.^8^ Pediatric early warning systems (PEWS) are bedside scoring tools associated with action algorithms that aid early identification of clinical deterioration in hospitalized pediatric patients.^9^ They have been shown to reduce incidence of hospital mortality in resource-limited settings^10^ and deterioration in all settings.^9^ PEWS have been evaluated in multicenter trials and validated in subspecialty populations, including pediatric oncology, across various resource settings.^11-13^ In addition, PEWS were recently emphasized as an international research priority to improve pediatric oncology care.^14^ Beyond quantitative effects on patient outcomes, qualitative work in a high-resource pediatric setting suggested that PEWS empower providers to overcome barriers to care escalation.^15^ Similar studies have examined how PEWS affect situational awareness and depend on interdisciplinary acceptance.^16^ To better understand these themes in pediatric oncology and resource-limited settings, we evaluated the impact of PEWS on interdisciplinary communication about patient deterioration in 2 pediatric oncology hospitals of different resource levels.

## METHODS

### Setting

This study was conducted at Unidad Nacional de Oncología Pediátrica (UNOP) and St Jude Children’s Research Hospital (SJCRH), 2 free-standing pediatric hematology-oncology hospitals with differing resources. The hospitals have similar missions, patient populations, and patient volume, and underwent a parallel process of PEWS implementation.^12,17^

UNOP is located in Guatemala City, Guatemala, an upper-middle-income country with a gross national income (GNI) of US $4,410 per capita and a childhood cancer survival rate of approximately 65%. UNOP treats 50% of Guatemalan children with cancer (500 new cancer diagnoses per year) and houses a 9-bed pediatric intensive care unit (PICU) with 300-400 admissions per year. Staffing includes 4 intensivists and approximately 1 oncologist for every 66 newly diagnosed patients per year. Nurse-to-patient ratios in the PICU and inpatient wards are 1:1-1:2 and 1:4-1:6, respectively. SJCRH is located in Memphis, TN, with a GNI of US $62,850 per capita and childhood cancer survival rate of > 80%. SJCRH treats 500 to 600 newly diagnosed cancer patients annually and has a 12-bed PICU with 350 to 400 annual admissions. SJCRH has > 40 treating oncologists, with approximately 1 oncologist for < 15 new diagnoses, and 8 intensivists. Nursing ratios in the PICU and inpatient wards are 1:1 and 1:2, respectively.

PEWS scoring tools and algorithms are similar at each site and involve bedside nursing assessments with routine measurement of vital signs. Elevated PEWS scores require medical team evaluation and, for high scores, PICU notification. At UNOP, the bedside nurse must call or physically locate the ward physician and PICU team to report an elevated PEWS score. A PICU consultation is recommended as part of this algorithm; however, there is no formal medical emergency response team at UNOP. At SJCRH, the bedside nurse calls the primary ward team to report an elevated PEWS score, and PEWS documentation initiates an automatic page and activates the rapid response team, which consists of a PICU nursing supervisor and a respiratory therapist who immediately evaluate the patient, and a mandatory PICU consultation within 30 minutes. If a patient needs urgent intervention or has a life-threatening emergency, the team can initiate a code, which activates immediate response from a larger medical team, including the PICU attending physician. Complete scoring tools and algorithms for each hospital are available in the Data Supplement. PEWS were implemented at UNOP in 2014^12^ and at SJCRH in 2016.^17^

### Participants

Participants from UNOP and SJCRH were purposively sampled from staff involved in the PEWS and care escalation, including bedside nurses and nursing coordinators, frontline physicians (ie, pediatricians and pediatric hematology-oncology fellows), and advanced practice practitioners (APPs; SJRCH only), and PICU providers (ie, attending physicians, fellows, and, at SJCRH only, APPs and intensive care nursing coordinators). Oncology attending physicians and health care assistants were excluded because they are not directly involved in the PEWS at either hospital. Eligible participants included providers involved in recent patient deterioration events, defined as an unplanned patient transfer from the inpatient ward to the PICU in the previous 8 weeks. The number of participants needed for thematic saturation, defined as the point at which no additional thematic information would be gained with additional interviews, was anticipated as 10-15 per discipline at each site.^18^ Interdisciplinary direct-care providers were recruited by study personnel not involved in institutional PEWS implementation or the deterioration event. Verbal consent was obtained; no identifying information was collected, and participants were asked to avoid using patient information. This study was approved by the ethics committee at UNOP and the SJCRH institutional review board.

### Study Design

This was a qualitative study using purposeful sampling and a grounded theory inductive approach to analysis. Trained researchers conducted semistructured interviews at SJCRH (J.S.G.) and UNOP (M.G.) during fall 2018. Interview questions and prompts formulated by the primary investigator (D.G.) were reviewed with research teams at both sites. Questions and prompts were written in English and translated into Spanish. The translation was reviewed by a team at UNOP for colloquial syntax and com-prehension, with feedback incorporated and revision as needed. Interdisciplinary team members piloted questions, with rewording and restriction as indicated prior to study initiation (final interview questions are provided in the Data Supplement). Interviews were conducted in person at each site, lasted approximately 25-30 minutes, were audio recorded, professionally translated, and transcribed for analysis. Twenty percent of transcripts were reviewed by a bilingual member of the team (M.G.) and compared line by line with audio recordings for accuracy.^19^ Transcripts were deidentified and further reviewed by primary investigator (D.G.) to assess thematic saturation.^20^

### Analysis

Transcribed interviews underwent qualitative content analysis using broad thematic domains derived inductively from transcript review.^21^ Three research team members iteratively read transcripts and identified potential codes on the basis of words used by participants and recurrent themes (M.G., G.F., D.G.). Each code was conceptually defined (see code definitions in the Data Supplement). Codes with overlapping meaning occurring together were combined, and broad or vague codes were divided. This process continued until consensus was reached and the codebook was finalized with definitions. Two researchers coded each transcript. Response to each question was the unit of response; a phrase or sentence was the unit of analysis, which was referred to as a segment. All segments were labeled with appropriate codes. Team members fluent in Spanish coded UNOP transcripts (D.G., M.G.) and returned to Spanish transcripts as necessary. After double-coding, transcripts were reviewed by a larger team including a third-party adjudicator (A.A.) to establish consensus and test interrater reliability (κ = 0.78). Coded segments were analyzed by reviewing themes independently. In addition to thematic content analysis, codes within certain domains (eg, resources or method of communication) were overlaid to evaluate intersections, relative im-portance, and complex communication dynamics. MAXQDA software (VERBI, Berlin, Germany) was used for data management and analysis. Consolidated Criteria for Reporting Qualitative Studies guidelines were followed to ensure rigor in analysis and qualitative data reporting.^22^

## RESULTS

A total of 42 and 41 interviews were conducted at SJCRH and UNOP, respectively. Table 1 describes the distribution of nurses, inpatient ward providers, and PICU providers interviewed at each institution. Content analysis revealed codes grouped into categories that described themes, including “resources,” “hierarchy,” “method of communication,” “quality of communication,” and “trigger.”

**TABLE 1:**
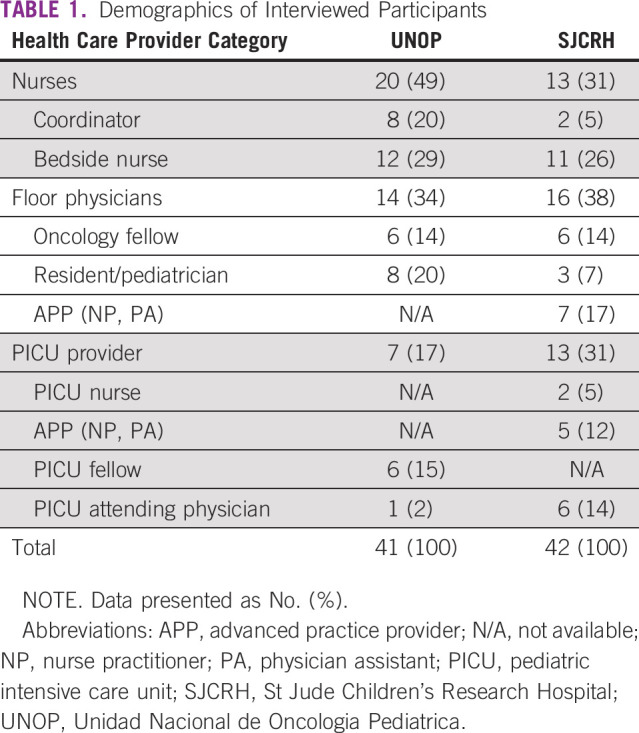
Demographics of Interviewed Participants

### Resources

Providers’ references to perceived resources were coded as “human resources,” “technology/infrastructure,” or “financial resources.” Resources were mentioned a similar number of times at both institutions (Table 2) and were not specifically identified as a barrier at UNOP. “Technology/infrastructure” was coded more frequently at SJCRH, where its role in communication was occasionally criticized: “I think part of it is how we communicate in this hospital…using pagers is antiquated” (PICU attending physician, SJCRH).

**TABLE 2:**
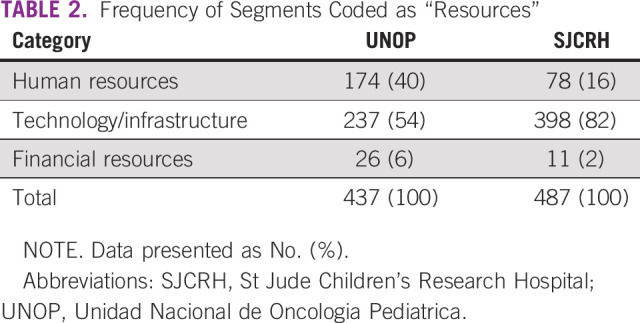
Frequency of Segments Coded as “Resources”

### Hierarchy and Empowerment

Themes related to team dynamics were coded as “reinforcement of hierarchy” and “empowerment.” All nursing interviews included these themes, with more coded segments in UNOP transcripts (Data Supplement).

Hierarchies were part of the underlying medical culture at both hospitals (Table 3). Bedside providers describe a ladder with PICU providers at the top, followed by ward providers, and nurses at the bottom. Hierarchy was identified as a persistent barrier to patient care, particularly at UNOP. Participants describe the PEWS as modifying this structure and changing institutional culture by empowering providers.

**TABLE 3:**
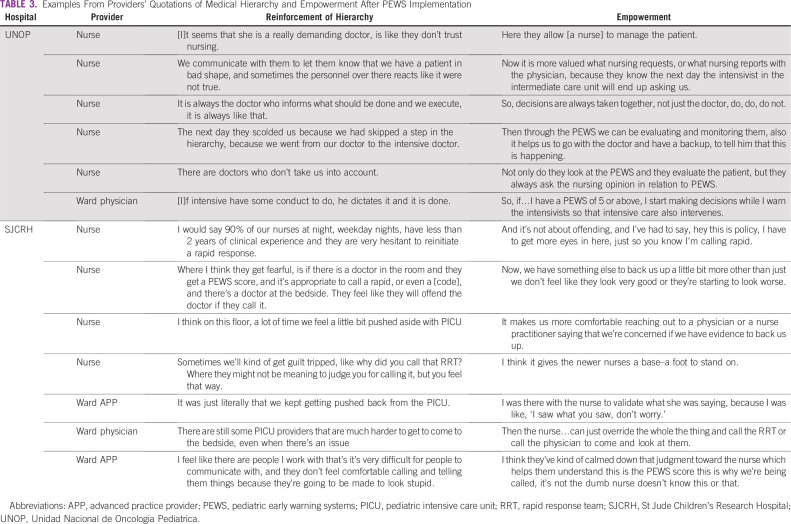
Examples From Providers’ Quotations of Medical Hierarchy and Empowerment After PEWS Implementation

Nurses at both institutions reported that the PEWS empowered them to clinically evaluate patients, make decisions, and talk to physicians (Table 3). Ward physicians and PICU providers noticed the same phenomenon: “I do think [PEWS] has given a stronger voice to nursing concern” (ward provider, SJCRH); “Everyone has the power to activate that alarm…be it nursing or the head nurse or a doctor” (PICU provider, UNOP).

Although some described empowerment, nurses and physicians at SJCRH also noted that the PEWS diminished clinical judgment: “They are so concerned with the data points and doing this routine task of assessment that it…dulls [nurses’] ability to consider those things when it is not time to do a [PEWS], or conversely, [nurses] lean so heavily on the quantitative data that [PEWS] presents versus appraising the patient situation” (PICU provider, SJCRH). They discussed that the PEWS limited nursing advocacy: “My gut had been ringing all day, but technically nothing had changed so…I can’t do anything about it” (nurse, SJCRH). Disempowerment, explored through the code “reinforcement of hierarchy,” was also described at UNOP but manifested as negative interpersonal interactions than as related to the PEWS: “We do not know if [our concern] will be taken into account”; “sometimes doctors are upset that one has more knowledge than them” (nurses, UNOP).

PEWS empowered ward physicians at UNOP to make decisions and act during a deterioration event: “If I have a [PEWS score] of 5 or above, I start making decisions while I warn the intensivists” (ward physician, UNOP). Occasionally, ward providers at SJCRH referenced empowered decisions to transfer deteriorating patients: “I texted the PICU…and said, ‘we’re sending this kid to the PICU’” (ward physician, SJCRH). However, recurring descriptions at SJCRH mention ward providers being excluded from communication rather than empowered by it: “I didn't actually know that the [rapid response team] was being called” (ward provider, SJCRH), often because the automated PEWS alerts were sent directly to PICU providers: “it doesn’t notify us…it is the nurse’s responsibility to let the primary team know” (ward provider, SJCRH). This gap in communication was not seen at UNOP.

### Communication: Method and Quality

Structural codes described who was communicating (eg, “nursing communication” was used for all conversations involving a nurse). Methods of communication included “in-person communication” and “phone or electronic communication.” Quality of communication was examined as “positive communication,” “negative communication,” or “lack of communication.”

Overall, providers described the PEWS as “a way to open communication” (nurse, SJCRH) and, at times, communication was the immediate response to an elevated score: “I see that the culture changed a lot…if the patient is not well, I instantly communicate” (nurse, UNOP). However, descriptions of communication quality varied: “There is more communication, I do not know if it is more cordial or not, but I think [PEWS] has forced us to have much more communication” (PICU provider, UNOP). Segments were coded as “positive communication” when descriptions were explicitly good: “I feel like the communication between everybody is better because of these scores” (PICU provider, SJCRH). Although there were no descriptions of PEWS worsening communication, references to “negative communication” included providers feeling ignored or reprimanded when reporting high scores and implied that interdisciplinary communication around the PEWS may exacerbate or reveal strained relationships: “If I am telling you the [PEWS] is a four or five and I want you to do something and you’re not doing anything [it] would cause me more anxiety” (ward provider, SJCRH); “Sometimes they scold us because the [PEWS] score” (nurse, UNOP).

Participants described the failure to communicate when necessary, which was coded as lack of communication. At UNOP, lack of communication related to personnel attitudes, team dynamics, or hierarchical culture: “There are doctors who are angry or serious, so that’s why [nurses] do not tend to tell them any signs or any alteration” (nurse, UNOP). At SJCRH, technology contributed to lacking communication: “You have no idea if they received it or if they ignored it or their pager is on or off” (PICU provider, SJCRH). Overlap between “technology/infrastructure” and “lack of communication” revealed 45 segments involving both codes. Other codes overlapping with “lack of communication” included “reinforcement of hierarchy” (53 segments) and “negative perception of a deterioration event” (56 segments; Data Supplement).

The character of communication (positive, negative, or lacking) was similar between institutions; however, the method of communication varied considerably. More “phone or electronic communication” was coded in interviews at SJCRH (62% of all segments within method of communication were phone or electronic). In contrast, more “in-person communication” was discussed by participants at UNOP (76% of all segments within the category), allowing for both positive and negative direct interpersonal interactions.

### Trigger

The PEWS were described as an alert or trigger for a deteriorating patient (Table 4). This code was observed in all interviews. Of 684 segments coded as “trigger,” 181 (26%) overlapped with a method of communication. There was more overlap between these codes at SJCRH (146 [35%] of 417 total trigger segments) than at UNOP (35 [13%] of 267 total trigger segments). At SJCRH, 78% of the overlap with “trigger” was “phone or electronic communication” (114 of 146 segments) due to automated alerts. In addition to communication, the PEWS were noted to trigger action and clinical judgment: “It allows us to detect early any alteration or complication that needs to be attended immediately” (nurse, UNOP).

**TABLE 4:**
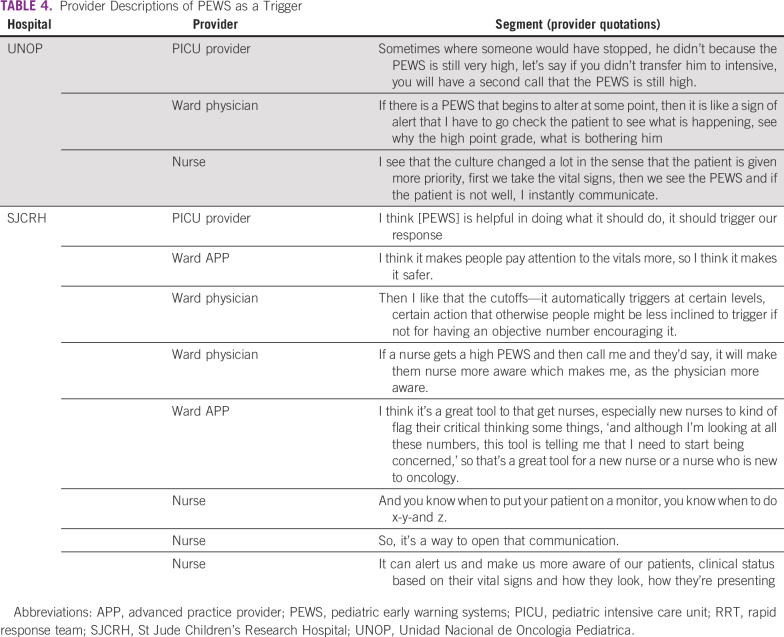
Provider Descriptions of PEWS as a Trigger

## DISCUSSION

PEWS were recently identified as the second most important research priority to improve outcomes of critically ill pediatric oncology patients.^14^ Although there is inadequate literature on using PEWS in resource-limited settings,^23^ previous studies demonstrate that PEWS implementation improves outcomes for children with cancer^24^ and using PEWS is a valid and cost-effective way to identify critical illness in these settings.^12,25^ Indeed, the impact of PEWS on patient outcomes may be greater in LMICs than in high-income countries because of higher baseline mortality rates and lower capacity for monitoring,^10^ including equipment shortages and lack of human resources such as medical emergency response teams. Studies in high-resource settings demonstrate PEWSs empower nurses despite hierarchical structures.^26,27^ Our study supports these findings and adds to the literature in resource-limited settings by demonstrating that PEWS empower both nurses and ward providers in pediatric oncology settings with varied resources.

Established medical hierarchies affected communication in both study settings. Participants described “negative communication” despite increased empowerment and more frequent interactions. In the high-resource setting, bedside-provider disempowerment was amplified by components of local PEWS use, including mandatory automated alerts that discouraged clinical judgement. These findings should motivate oncology teams implementing interventions that affect frequency of communication, such as PEWS, to consider methods of provider communication and the effect increased communication may have on existing interdisciplinary relationships and power dynamics.

Implementation processes and decisions may also affect how PEWS are used and may explain some of the differences seen between these institutions. For example, the lack of a rapid response system and less automation in the resource-limited setting seems to have encouraged in-person communication, contributing to positive interaction and collaborative patient care. In settings of all resource levels, combining PEWS implementation with education on communication and interdisciplinary team dynamics may maximize impact on patient care. Potential examples include interdisciplinary PEWS teams made up of nursing and physician leads, quality-improvement interventions aimed at communication, and interactive or case-based role play focused on elements of team communication, including respect.^28,29^ Targeted communication education may be particularly useful in resource-limited settings, where oncology providers receive little to no standardized communication training.^30^ Notably, neither financial nor technologic resources were perceived barriers to care escalation in either setting. This finding supports system-level improvements that capitalize on existing infrastructure and endorses use of PEWS in pediatric oncology settings of all resource levels.

This study has several limitations. It was conducted at 2 institutions and the results may not be transferable beyond these settings. Interviews were conducted in 2 languages, with analysis in English, which increased consistency but may have slightly changed the interpretation of original statements. To minimize misinterpretation, a bilingual member of the study team confirmed translation, and Span-ish transcripts were retained and reviewed as needed.^19^ We attempted to limit social desirability bias by using study personnel outside of the institution’s clinical care setting; however, it is unknown if this was successful. Finally, the PEWS functionality and participant perception may have been affected by differences in institutional algorithms, culture, and local education and training.

Overall, interdisciplinary pediatric oncology providers working within differently resourced institutions agreed that PEWS empower providers to start essential conversations and trigger medical interventions in deteriorating patients. However, elements of underlying medical culture (hierarchy), team dynamics (negative communication), technology (contributing to electronic or lacking communication), and automation (leading to diminished clinical judgment) may decrease the impact of PEWS on patient care. Potential barriers to interdisciplinary communication should be evaluated and addressed before and after PEWS implementation. In hospitals already using a PEWS, frequent evaluation of barriers is likely to improve interdisciplinary communication and maximize impact on patient care. Finally, this study emphasizes how qualitative analysis can be combined with traditional quantitative clinical outcome measures in the assessment of quality-improvement efforts and encourages additional research on interdisciplinary communication in pediatric oncology settings of all resource levels.
